# The role of interlayer adhesion in graphene oxide upon its reinforcement of nanocomposites

**DOI:** 10.1098/rsta.2015.0283

**Published:** 2016-07-13

**Authors:** Zheling Li, Ian A. Kinloch, Robert J. Young

**Affiliations:** School of Materials and National Graphene Institute, University of Manchester, Oxford Road, Manchester M13 9PL, UK

**Keywords:** graphene oxide, nanocomposites, mechanics, Raman spectroscopy

## Abstract

Graphene oxide (GO) has become a well-established reinforcement for polymer-based nanocomposites. It provides stronger interfacial interaction with the matrix when compared with that of graphene, but its intrinsic stiffness and strength are somewhat compromised because of the presence of functional groups damaging the graphene lattice and increasing its thickness, and its tendency to adopt a crumpled structure. Although the micromechanics of graphene reinforcement in nanocomposites has been studied widely, the corresponding micromechanics investigations on GO have not been undertaken in such detail. In this work, it is shown that the deformation micromechanics of GO can be followed using Raman spectroscopy and the observed behaviour can be analysed with continuum mechanics. Furthermore, it is shown that the reinforcement efficiency of GO is independent of its number of layers and stacking configurations, indicating that it is not necessary to ensure a high degree of exfoliation of GO in the polymer matrix. It also demonstrates the possibility of increasing the concentration of GO in nanocomposites without sacrificing mechanical reinforcement efficiency.

This article is part of the themed issue ‘Multiscale modelling of the structural integrity of composite materials’.

## 1. Introduction

Graphene-related materials are promising candidates as reinforcements in polymer nanocomposites owing to their combination of high modulus and strength [1]. Additionally, their wide range of other impressive physical properties makes it possible to develop multifunctional nanocomposites [1,2]. The deformation mechanics of pristine graphene has been extensively studied, both in the free-standing state [3] and during reinforcement in nanocomposites [1,4,5]. In particular, it has been shown, by using the strain-sensitive shifts of the Raman bands, that the deformation of graphene follows continuum mechanics developed for macroscale fillers [6–8], and the interfacial shear stress with a polymer matrix is of the order of 2 MPa [5,9]. However, graphene is hard to disperse homogeneously in a polymer matrix because of its tendency to agglomerate and the poor compatibility of its chemically inert surface with the matrix. Additionally, for multilayer graphene, its reinforcement efficiency reduces as a result of poor interlayer stress transfer [10,11], which can further cause a loss of the Bernal stacking in the multilayer graphene that is regained upon unloading [12]. In contrast, the presence of functional groups on its derivative, graphene oxide (GO), give a good degree of matrix–reinforcement interaction leading to improved dispersion and stress transfer [13]. However, the presence of the functional groups in the GO both damages the graphene lattice and increases flake thickness, leading to a loss of intrinsic stiffness by a factor of approximately 4 [14]. These competing factors strongly suggest that there may be an optimal degree of functionalization that gives sufficient matrix interaction without significantly reducing the stiffness.

The Young modulus of monolayer GO has been shown to be of the order of 250 GPa [14]. A computer simulation estimated a maximum interfacial shear stress for a GO nanocomposite to be more than 130 MPa at the edges of reduced GO flakes with a size of approximately 30 μm [15], significantly higher than the maximum interfacial shear stress for graphene [5,9]. GO-based paper has also been investigated and it displays a good layered structure that endows exceptionally high stiffness [13]. Although the deformation mechanisms of monolayer GO and its impregnated papers have been studied extensively [13,16–19], a comparison implies that the factors that affect the stiffness of GO-based materials differ significantly with length scale [20]. Even more importantly, as an intermediate material between monolayer GO and GO paper, few-layer GO (number of layers less than 20) and multilayer GO (number of layers over 20) have been studied less extensively regarding their reinforcement of bulk nanocomposites. In particular, the deformation/reinforcement mechanics of GO with different number of layers has not been studied systematically. This may be due to the difficulty in fabricating nanocomposites reinforced solely with mono- or few-layer GO flakes. In addition, it is also not easy to manipulate the complicated stacked GO structure in micromechanical testing instruments. Despite these difficulties, few-layer GO could, in fact, have even wider application: (i) it is easier to prepare than monolayer GO and is thus more amenable to scale-up; (ii) it is more likely to be present than monolayer GO in some nanocomposite matrices, especially hydrophobic polymers, or even ceramics and metals; and (iii) bulk nanocomposites based on stacked GO have more practical applications than GO paper.

In this work, the deformation mechanics of mono-, bi- to multilayer GO structures with different stacking configurations have been studied. The shift of the Raman D band position (*ω*_D_) of GO as a function of strain *ε* has been used to follow the deformation mechanics of monolayer GO flakes to investigate the validity of continuum mechanics on the microscale. Based on the estimation of the effective Young modulus of GO [21], its shift rate with strain (d*ω*_D_/d*ε*) is further used to compare the reinforcement efficiency of a series of different GO flakes.

## 2. Experimental

### (a) Materials

The graphite (grade 2369) was supplied by Graphexel Ltd. All other reagents were of analytical grade and used without further purification.

### (b) Preparation

The GO was prepared using the modified Hummers method, and the details of preparation can be found elsewhere [21–23]. After graphite was oxidized, the GO was dispersed in water, diluted to less than 10^−3^ mg ml^−1^ and deposited onto poly(methyl methacrylate) (PMMA) beam followed by drying under ambient conditions. Prior to the GO deposition, the PMMA beams were treated using a UV–ozone plasma, facilitating the spreading of the GO dispersion and also the adhesion of the GO to the substrate.

### (c) Characterization

Transmission mode Fourier-transform infrared (FTIR) spectrum was obtained from dried GO powder mixed with KBr, using a Nicolet 5700 spectrometer (ThermoFisher Scientific Inc.). X-ray diffraction (XRD) was carried out on dried GO powder using an X’Pert DY609 X-ray diffractometer (Philips) with a Cu-K*α* radiation source (λ=1.542 Å). Atomic force microscope (AFM) images were obtained using a Dimension 3100 AFM (Bruker) in the tapping mode in conjunction with a ‘TESPA’ probe (Bruker).

Raman spectra were obtained using Renishaw 1000/2000 spectrometers and a Horiba LabRAM HR Evolution spectrometer equipped with HeNe lasers (λ=633 nm) with laser spot sizes of around 1–2 μm. The incident laser polarization was parallel to the strain, whereas the scattered radiation was randomly polarized. The specimens were deformed in a four-point bending rig, and the strain was measured using a strain gauge placed close to the region being analysed [24].

## 3. Results and discussion

### (a) Microstructure

**Figure 1.**
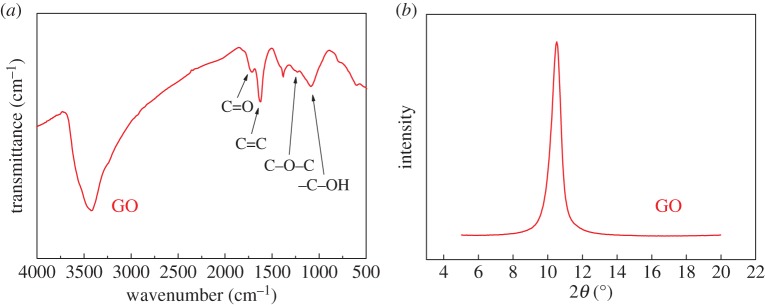
(*a*) FTIR spectrum and (*b*) XRD pattern for the GO powder.

The FTIR spectrum of the GO is shown in figure 1*a*, where a number of absorption bands, corresponding to the presence of functional groups, can be observed. For example, the band around 1710 cm^−1^ can be attributed to the C=O stretching of the ketonic species, and the band around 1620 cm^−1^ is assigned to alkene in the graphitic region. The epoxy C–O–C bonds generate a band at around 1230 cm^−1^, whereas the band resulting from the C–OH stretching is located around 1070 cm^−1^ [25]. These functional groups were introduced after oxidation [26] and expand the interlayer separation, as confirmed using XRD (figure 1*b*), where the characteristic peak for the GO at 2*θ*=10.5° corresponds to an interlayer spacing of 0.84 nm, in good agreement with the reported value [16].

### (b) Deformation of monolayer graphene oxide

**Figure 2.**
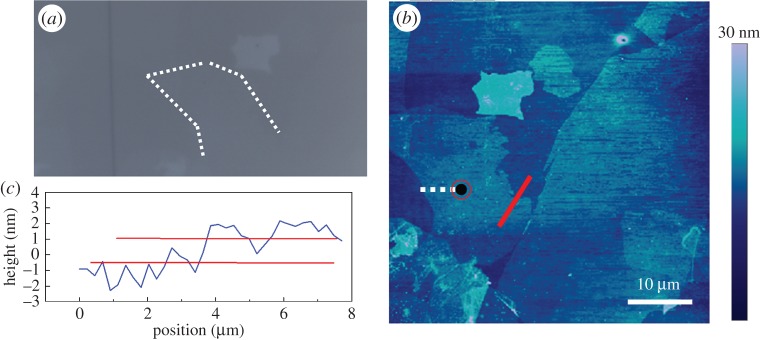
(*a*) Optical microscope image of the GO flake, and the dashed line highlights its edge. (*b*) AFM image of the GO flake, where the black spot indicates where the deformation was monitored. (*c*) Height profile along the red line in (*b*), and the red lines in (*c*) are guides to the eyes showing the thickness of the flake.

As reported previously [1,4,5,9], the deformation of graphene can be followed using Raman spectroscopy. The small spot size of the Raman laser enables the spatial resolution of the measurement down to micrometre dimensions, thus revealing the local strain distribution accurately in individual flakes. However, unlike the Raman spectrum of graphene where the number of layers can be identified through a fingerprint spectrum [5,27], the Raman spectra of GO are very similar, regardless of the number of layers. This makes the analysis very difficult especially with GO flakes on the top of a polymer substrate. Hence, AFM was used here as a supplementary technique to find, identify and characterize the GO flakes (figure 2). It was carried out in tapping mode, so that no damage was induced in the structure of the GO, as confirmed in §1 of the electronic supplementary material. It can be seen that the GO flake in figure 2 has a lateral dimension of over 15 μm, and the height profile obtained clearly shows it has a thickness of the order of 1 nm, demonstrating its monolayer nature (figure 2).

**Figure 3.**
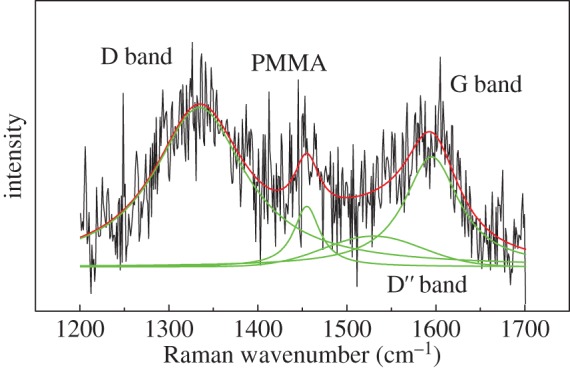
The Raman spectrum of the GO on PMMA (black) with the fitted curved (red) and also the fitted curved for each band (green).

A Raman spectrum was then obtained from this typical monolayer GO flake as shown in figure 3. The most prominent bands in the spectrum are the G band and D band. The G band, located at approximately 1600 cm^−1^, is due to the doubly degenerate E_2g_ vibrational mode at zone centre [27]. The D band, centred at approximately 1330 cm^−1^, results from the A_1*g*_ symmetry *K* point phonons [28]. The small band around 1450 cm^−1^ is from the PMMA substrate. The broadening of the D and G bands compared with those of graphene [27] is possibly due to the loss of lattice symmetry as a result of the breaking down of the sp^2^ carbon networks of pristine graphene by the strong oxidation [28,29], implying a higher degree of disorder [30,31]. This also explains the weaker signal and more scattered data points than those of graphene [1], because the damaged graphene hexagonal lattice leads to a loss of the resonance Raman scattering. Both D band and G band were fitted with a single Lorentzian peak, as shown in figure 3. The value of the ratio of the intensity of the two bands, *I*_D_/*I*_G_, can actually be used as a measure of the degree of disorder of GO [30,32]. It should be noted that a D^′′^ band (approx. 1520 cm^−1^) also needs to be considered, with a Gaussian function fitting needed to account for the broad shoulder between the D and G bands, which may be linked to the structural disorder of the GO [33,34]. Even though another D’ band around 1620 cm^−1^ is present [19,33], the curve fitting employed is still thought to be a good first approximation considering the spectral resolution and disordered structure.

Another issue for concern is that GO, being chemically different from graphene because of the functional groups [35], may make it more vulnerable to damage under the Raman laser beam, where it may tend to undergo, for example, heat reduction [36] or photoreduction [37]. To avoid this, a laser with a relatively long wavelength (λ=633 nm) was used in this work because of the absorption peak of GO in the higher energy region near approximately 250 nm [36]. Local laser heating was also avoided by using low laser power. The lack of beam damage was indicated by the constant value of *I*_D_/*I*_G_ after four/five scans on both monolayer and bilayer GO flakes (electronic supplementary material, figure S2) [30,32].

**Figure 4.**
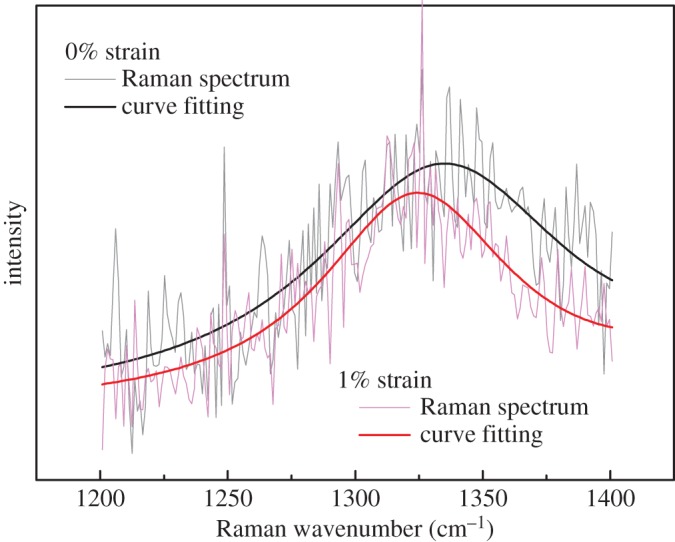
The Raman D band of the GO flake before (grey) and after (pink) being deformed to 1.0% strain. The black and red curves, respectively denote the curves fitted as shown in figure 3.

**Figure 5.**
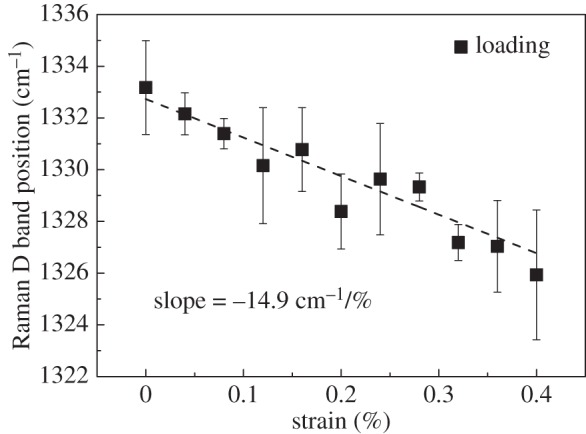
The Raman D band position *ω*_D_ as a function of strain of the GO flake, measured at the point in figure 2.

The GO was then deformed stepwise and Raman spectra were collected at each strain level. The Raman D band was used to follow the deformation of the GO [37]. This band was preferred to the normal Raman bands used to follow deformation in graphene [38], the two-dimensional band [32] which is weak and broadened in GO and the G band [19] which is asymmetric. The D band was used to monitor the deformation of a point in the central region of the flake (figure 2), where good stress transfer should take place [5]. A clear downshift of *ω*_D_ can be seen during deformation of the GO to 1.0% strain (figure 4), as a result of the elongation of the C–C bonds during deformation [8,39]. The variation of *ω*_D_ with strain determined at the point shown in figure 2 was used to follow the deformation of the GO flake *in situ* (figure 5). An approximately linear downshift with strain was observed as the GO was deformed to 0.4% strain, with a slope d*ω*_*D*_/d*ε* of −14.9 cm^−1^/%. This was the result of the elastic stress transfer from the substrate to the GO. The slope d*ω*_*D*_/d*ε* decreases somewhat at a higher strain as can be seen in electronic supplementary material, figure S3, perhaps indicating partial interfacial failure [5,9,40], or a variation of Grüneisen parameter with strain [41].

From the knowledge of the Grüneisen parameter [8,39,42], it has been demonstrated that, for an ideal graphene crystal, d*ω*_D_/d*ε* under uniaxial tensile strain should be related to it through a relation of the form [8,43]
3.1
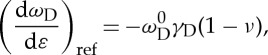
where *ω*^0^_D_ is *ω*_D_ at zero strain, *γ*_D_ is the Grüneisen parameter for the D band and *ν* is the Poisson ratio of the PMMA substrate. Based on equation (3.1), if a value of *γ*_D_=3.55 is used [8], and *ν* is taken as 0.35 for the PMMA substrate [44], the reference value of d*ω*_D_/d*ε* ((d*ω*_D_/d*ε*)_ref_) for supported graphene should be of the order of −30 cm^−1^/%.[8] The value obtained here is just approximately half of that for graphene [8,43], probably due to the structural distortion resulting from the sp^3^ hybridization of carbon atoms [45] and waviness or crumples [40]. These factors could easily decrease the value of d*ω*_D_/d*ε* for GO by a factor of 2 [46] and the value of d*ω*_D_/d*ε* determined here is in good agreement with the value estimated during the deformation of a variety of nanocomposites [47]. (The value of d*ω*_D_/d*ε* in reference [47] was calculated for free-standing GO excluding the effect of Poisson ratio, while here it is for supported GO.)

At higher strain levels (electronic supplementary material, figure S3), the value of d*ω*_D_/d*ε* decreases, corresponding to less-efficient interfacial stress transfer [5,9,40], or a dependence of the Grüneisen parameter on strain [41]. This less-efficient stress transfer may be due to the intrinsically weak bonding between PMMA and GO [48]. There is, however, still stress transfer taking place, probably due to the friction [9] as a result of the interaction between the functional groups and the substrate and also the rough morphology of the GO surface [49].

### (c) Deformation mechanics of monolayer graphene oxide

**Figure 6.**
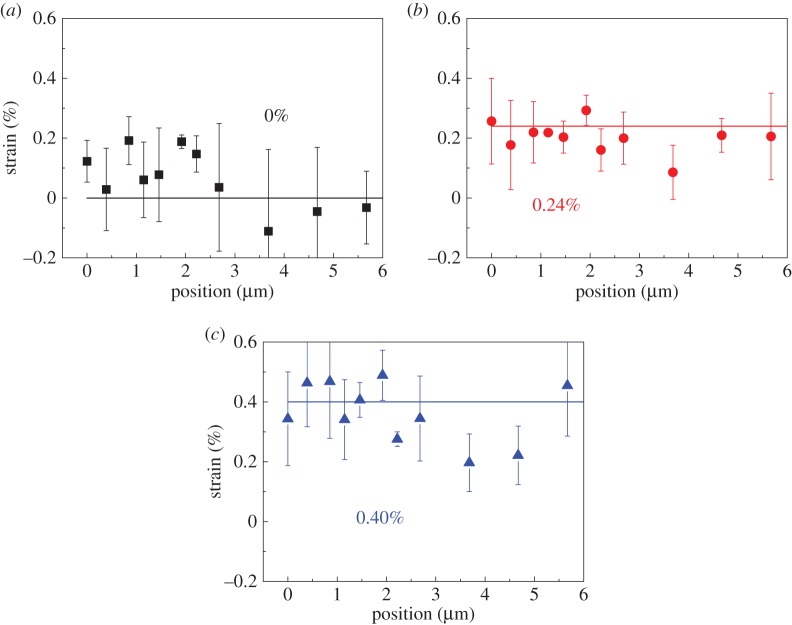
The strain distribution of the GO flake at a strain of (*a*) 0%, (*b*) 0.24% and (*c*) 0.40%. (Online version in colour.)

Similar to our earlier studies on monolayer pristine graphene [1], Raman spectra were collected from different points going from the edge of the GO flake to its central region, as indicated by the dashed white line in figure 2. This was done for different levels of strain applied to the PMMA beam. The linear fit in figure 5 can be used as a calibration to correlate *ω*_D_ with local value of strain *ε* in the GO flake [1,4,5,9]. It can be seen in figure 6 that, before deformation, the strain distribution from the GO flake edge to the centre was approximately 0%. When the PMMA beam was deformed to 0.24% and then to 0.40% strain, the strain in the central region of the GO flake increased uniformly, with the strain in the GO being similar to that of the PMMA beam, confirming good interfacial stress transfer from the substrate to the central region of the GO flake, as has been found before for graphene [5,9].

There is, however, a major difference between the behaviour of GO and graphene near the flake edge. The deformation mechanics of graphene and GO can be described using the well-known ‘shear-lag’ theory (§4 in electronic supplementary material). However, in this work, within the scatter in the data, the strain distribution appears to be constant with position along the flake, whereas in the case of pristine graphene, the strain falls over several micrometres to zero near the edge of the flake due to shear lag effects, enabling the ‘critical length’ of the graphene for good reinforcement to be determined [5,9,50]. This could be partially due to the weak Raman scattering from GO, and also the difficulty to clearly identify the poorly resolved GO edge under the optical microscope (figure 2). It does, however, also suggest a shorter ‘critical length’ compared with that of graphene. Another factor that needs to be taken into account is the spatial resolution of the Raman laser (1–2 μm), which means that values of ‘critical length’ around 1 μm will be difficult to measure [51]. In any case, the data still clearly imply that the critical length for GO is very short, hence in figure 6 only straight lines were drawn instead of curves derived from the ‘shear-lag’ analysis, for simplicity. This is a result of the presence of functional groups in the GO, and thus implies that, in nanocomposites, the interaction formed between GO and the polymer matrix especially when processed *in situ*, such as for epoxy resins, will lead to a stronger interface. It also explains the higher measured values of strength and toughness of GO nanocomposites than for nanocomposites reinforced with less functionalized graphene-based materials [52,53]. Further effort is still needed for a clearer identification of the strain distribution at the edge of a GO flake.

### (d) Deformation of few-layer graphene oxide

**Figure 7.**
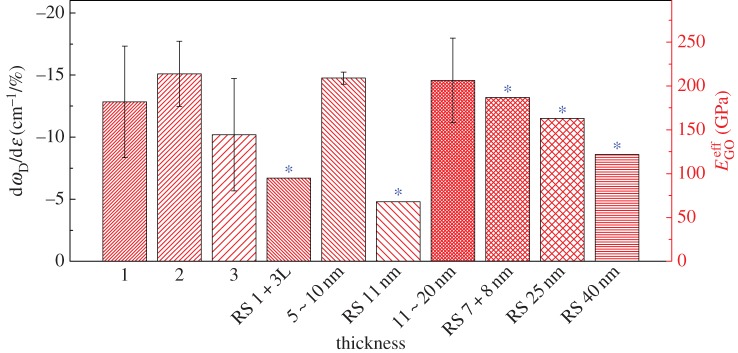
The Raman D band shift rate d*ω*_*D*_/d*ε* (left) and *E*^eff^_GO_ (right) of GO flakes for different layer number/stacking configurations. (Online version in colour.)

It has been shown that in pristine graphene with no functional groups the reinforcement efficiency decreases as the number of layers increases [11], as a result of the poor interlayer stress transfer [10]. Following the analysis on monolayer GO, it was therefore instructive to compare its behaviour with that of GO with different numbers of layers and stacking configurations. Model GO configurations have also been fabricated on PMMA beams with different number of GO layers, ranging from mono-, bi-, tri- to multilayer (denoted as *X*L, where *X* is the number of layers, for multilayers denoted as its thickness in nm, assuming for simplicity that the thickness of the GO monolayers is approximately 1 nm), which were again identified using AFM. Their corresponding values of d*ω*_D_/d*ε* are shown in figure 7, and it is seen that, even though the number of layers varies, the value of d*ω*_*D*_/d*ε* does not change significantly.

**Figure 8.**
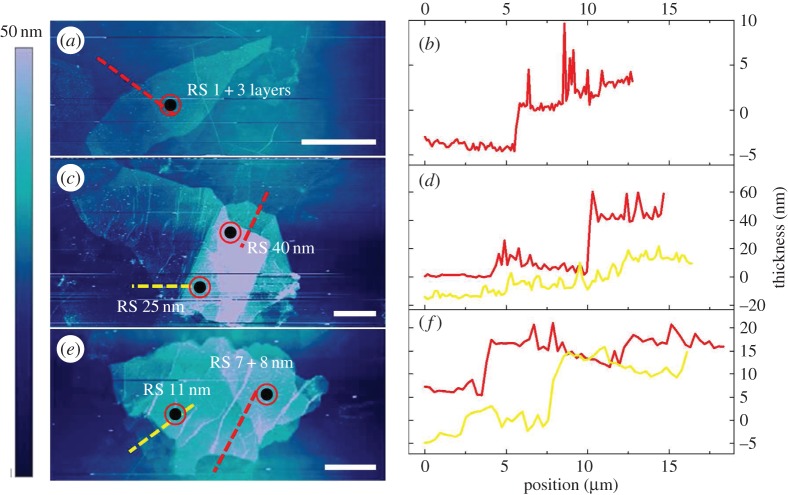
(*a*,*c*,*e*) AFM images of the re-stacked GO flakes where *in situ* Raman analysis was carried out at the highlighted points. Scale bar, 10 μm. (*b*,*d*,*f*) The height profiles corresponding to the dashed lines with the same colour in (*a*,*c*,*e*), respectively.

As well as the number of layers, what is notable is the stacking configuration of the GO flakes. Some of the GO flakes display a stacked multilayer structure, whereas others have a re-stacked structure (prefixed as RS before the thickness as in figure 7; for example, RS 1+3L means the re-stacked mono- and tri-layers; the full definition is shown in §5 in the electronic supplementary material), formed probably during the deposition of the GO on the PMMA substrate. Regardless of this difference, however, the values of d*ω*_D_/d*ε* are still similar as highlighted with stars in figure 7. Several of the re-stacked GO flakes that were tested are shown in figure 8, where it can be seen that they have re-stacked after being exfoliated.

### (e) Reinforcement efficiency of graphene oxide

It has been demonstrated previously that the value of d*ω*_D_/d*ε* can be used to estimate the effective Young modulus of GO (*E*^*eff*^_GO_) in nanocomposites [37]. The average value of d*ω*_D_/d*ε* measured in this present study was approximately −12.8 cm^−1^/% strain for monolayer GO. The effective Young modulus of GO compared with pristine graphene can be calculated from the reduced value of d*ω*_D_/d*ε* due to lattice damage and the increase in thickness as a result of the oxidation of the GO using the equation
3.2
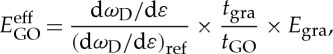
where (d*ω*_D_/d*ε*)_ref_ is the D-band shift rate for graphene (approx. −30 cm^−1^/%), *t*_gra_ is the thickness of graphene (0.34 nm), *t*_GO_ is the thickness of GO measured in this present study and *E*_gra_ is the Young modulus of graphene. This equation yields a value of *E*^eff^_GO_∼180 GPa for the GO, in broad agreement with but slightly lower than the value reported for an indentation test on a GO membrane [14]. This slightly lower value might be the result of the lack of strong adhesion between the GO flakes and the PMMA substrate.

**Figure 9.**
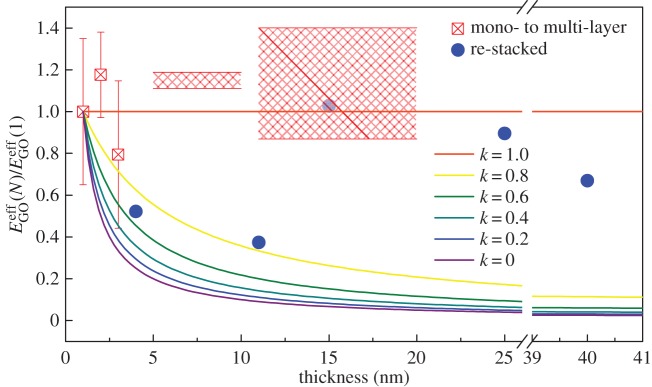
Values of 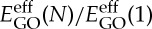 as a function of thickness of the GO flakes. The interlayer stress transfer efficiency factor *k* is taken as different values as shown in coloured lines. The shadowed regions represent a range of *k* values in the corresponding thickness range.

As the number of GO layers increases, up to tens of layers, the *E*^eff^_GO_ of GO of *N* layers (*E*^eff^_GO_(*N*)) derived from the Raman band shift data (figure 7) still does not change significantly compared with the rapidly decreasing value for graphene [11]. This phenomenon implies a similar stiffness of GO, regardless of the number of layers. It can be attributed to the good interlayer adhesion between the GO flakes, at least up to the number of layers evaluated. In order to study the interlayer stress transfer efficiency, a model originally developed for carbon nanotubes [54,55,56] and then for model graphene nanocomposites can be modified for this present study as [11]
3.3
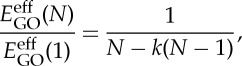
where the *E*^eff^_GO_(*N*) values are calculated from equation (3.2), and *k* is the interlayer stress transfer efficiency factor, ranging between 0 and 1. As all the GO flakes will have the same contact with the PMMA substrate, the value of *E*^eff^_GO_(1) is used as the reference in equation (3.3), the *k* worked out only reflects the interlayer adhesion of GO instead of the interface with PMMA substrate. The calculated *E*^eff^_GO_(*N*)/*E*^eff^_GO_(1) is plotted as a function of the number of GO layers with a series of values of *k* in figure 9, represented by the coloured lines. In order to count the number of layers, the thickness of monolayer GO was taken as 1 nm for simplicity. In this situation, if *k*=1, *E*^eff^_GO_(*N*)/*E*^eff^_GO_(1)=1, the stress can be transferred completely through the bottom layer to the top layer, thus the effective modulus of GO would be identical, regardless of the number of layers. For all the other values of *k*, the ratio of *E*^eff^_GO_(*N*)/*E*^eff^_GO_(1) decreases as the number of layers increases, in accordance with a gradual loss of the interlayer stress transfer from layer to layer. Particularly, for *k*=0, *E*^eff^_GO_(*N*)/*E*^eff^_GO_(1)=1/*N*, only the bottom layer bears the load, and no stress transfer occurs to the upper layers, hence the effective modulus varies inversely with the number of layers (cross-section area). It can be seen in figure 9 that most of the data points, except two, obtained in this work fall around the line where *k* equals unity, suggesting almost complete interlayer stress transfer in the GO flakes, regardless of the different number of layers and stacking configurations.

This behaviour of GO is remarkably different from that of graphene [11], for which its chemically inert surface makes interlayer sliding relatively easy, certainly in a multilayer graphene crystal [11,12] and probably also for restacked material. In contrast, GO behaves more like a ‘solid’ material, where the layers are strongly bonded to each other and interlayer sliding is less likely to occur, at least in the thickness range studied in this work. The finding here suggests a comparable Young modulus of few-layer GO to that of monolayer GO. This is well supported by the previous conclusion that the Young modulus of GO membranes scales with their number of layers, when assuming a constant thickness. If a thickness change is considered, it actually suggests the independence of the Young modulus upon the number of GO layers [14].

This behaviour can be interpreted as being due to the hydrogen bonding controlled by the functional groups and also the water content [57], which therefore leads to a strong interlayer adhesion [20]. This also explains why the few-layer GO fractures due to an intraplanar failure mechanism rather than a ‘pull-out’ mechanism when subjected to deformation [20]. Nevertheless, as we found and also reported in the literature, this situation is probably valid only up to tens of nanometres [20]. As the length scale increases to bulk materials such as GO-impregnated papers, other effects such as defects and voids [58], misorientation [47] and waviness [40] of the GO will inevitably become dominant and will affect mechanical properties, whereby ‘interplanar’ failure mechanisms start to appear [20,58]. Hence, a dependence of the Young modulus of GO paper upon its thickness has been found on the macroscopic scale [58].

In our samples, the GO forms after being oxidized and further separates in aqueous solution [26,59]. The remaining 2.2 wt% sulfur (as measured in elemental analysis) or water [35] can result in cross-link bonding between neighbouring GO layers that prevents them being separated even after oxidation [26,59]. This is similar to the previous microscopic observation that two functionalized GO flakes with identical geometries stick together even after oxidation [59]. This is evidenced by the constant measured value of d*ω*_*D*_/d*ε*, which would otherwise be expected to vary depending on the number of layers, as for graphene [11], where only the weak van der Waals forces act [10,11,12], when it had not been completely exfoliated. Even after the GO flakes were separated in water, the strong interlayer adhesion can be recovered after re-stacking. This is also the result of the water molecules being introduced during sample preparation that help to re-interconnect the separate GO flakes, giving interlayer adhesion comparable to that of the non-exfoliated counterparts.

These features of stacked and re-stacked GO flakes can be instructive for their use in GO-based nanocomposites. First, because of the strong interlayer adhesion, it is thought that there is less need to ensure a complete exfoliation of GO (less than 40 layers should suffice). As it is the GO flakes with large lateral dimensions that take longer times to be fully exfoliated [26], this removes the necessity for complete exfoliation, allowing the moderate treatment, which in return avoids the damage of the lateral flake dimension, thus giving better reinforcement [5,60]. Second, the strong interlayer adhesion recovered after re-stacking justifies the use of nanocomposites with high GO loadings without sacrificing the potential loss of mechanical performance due to GO re-aggregation [48]. It is expected that an even better interlayer adhesion might be obtained through further functionalization of GO [52,57].

## 4. Conclusion

This work has presented a method to monitor the deformation mechanics of GO with different number of layers and stacking configurations using Raman spectroscopy. The stress/strain-sensitive Raman D band was used, and the measured value of the shift of the position of the Raman D band with strain, d*ω*_D_/d*ε*, has been used to map the deformation of a monolayer GO flake, so that the strain distribution of the GO flake could be revealed. Additionally, the measured value of d*ω*_D_/d*ε* has also been used to estimate the effective Young modulus *E*^eff^_GO_ of GO flakes with different thickness. It is found to be almost constant, regardless of the number of layers or the stacking/re-stacking configuration. This was attributed to the good interlayer stress transfer provided by hydrogen bonding between the layers. It suggests that there is no need to exfoliate the GO completely when preparing nanocomposites. This also has the advantage of enabling flakes with relatively large lateral dimension to be prepared which will still have a significant positive impact on the reinforcement. Furthermore, it shows the possibility of preparing GO-based nanocomposites with a reasonably high GO content, without sacrificing mechanical performance due to re-aggregation effects.

## Supplementary Material

Supporting Information

## Supplementary Material

Original Data Files

## Data accessibility

The datasets supporting this article have been uploaded as part of the electronic supplementary material.
