# Supplementary material for: The role of interlayer adhesion in graphene oxide upon its reinforcement of nanocomposites
Source: Philos Trans A Math Phys Eng Sci. 2016 Jul 13;374(2071):20150283. doi: 10.1098/rsta.2015.0283 (PMC4901251; doi:10.1098/rsta.2015.0283)
Supplement: Supporting Information [file rsta20150283supp1.doc]

**The Role of Interlayer Adhesion in Graphene Oxide upon Its Reinforcement of Nanocomposites (Supporting Information)**

## 1. Damage

An optical microscope and an AFM were used to identify monolayer GO (Supplementary Figure 1). The optical image is in fact poorly resolved but a change of contrast at the GO edge is still visible, as highlighted in Supplementary Figure 1(a). Its geometry is much more clearly revealed by the AFM image (Supplementary Figure 1(e)), and it can be seen that the GO flake has a large lateral dimension, and is generally flat but with some wrinkles at its edge. The damage of the AFM tip to the GO flake was studied by scanning repeatedly over a small area followed by a global scan on the whole flake (Supplementary Figure 1(b), (c), (d) and (e)). The almost identical height profiles (Supplementary Figure 1(f)) over the dashed white lines in Supplementary Figure 1(b), (c), (d) and (e) obtained in each cycling scan imply that the tapping mode AFM scan induces no damage and the structure of the GO flake remains intact. Additionally, these height profiles clearly demonstrate the monolayer feature of the GO flake with a thickness of the order of 1~2 nm (Supplementary Figure 1(f)) .

**Supplementary Figure 1** (a) Optical image of the monolayer GO. AFM images of the (b) 1^st^, (c) 2^nd^, (d) 3^rd^ cycle of the local area of the GO flake and (e) the overall scan (4^th^ cycle) over the whole GO flake. (f) Height profiles along the dashed lines in (b), (c), (d) and (e), respectively. The surface of GO flake and the PMMA substrate underneath are highlighted with the dashed red lines.

## 2. Laser Heating Damage

Monolayer and bilayer GO flakes were scanned four to five times when they were strain-free, and the constant values of intensity of the Raman D band divided by the intensity of the G band, *I*_D_/*I*_G_ imply the absence of any reduction induced by the laser heating (Supplementary Figure 2) .

**Supplementary Figure 2** The Raman band intensity ratio, *I*_D_/*I*_G_ values of monolayer (red) and bilayer (blue) GO during four/five scans.

## 3. Loading and Unloading of the GO Monolayer

The value of d*ω*_D_/d*ε* flattens at a higher strain, perhaps indicating a partial interfacial failure , or a variation of Grüneisen parameter . When the sample was unloaded, *ω*_D_ increased back towards its original position. This reversible behaviour, to some extent, suggests elastic deformation . Additionally, it also demonstrates that the downshift of *ω*_D_ is due to the deformation of the GO rather than any laser-induced heating effect.

**Supplementary Figure 3** The Raman D band position, *ω*_D_ as the function of strain of the GO flake, measured on the point in Error: Reference source not found.

## 4. Shear-lag analysis on the GO flakes

Similar to graphene , the deformation mechanics of GO flake can be described using the ‘shear-lag’ analysis , where the stress is transferred from the substrate to the filler through shear stress and the strain increases from edge of the filler until a plateau in the centre. The real strain *ε*_r_ of the GO flake can be given as:

$\varepsilon_{\text{r}}=\varepsilon_{\text{m}}\left[1-\frac{\cosh\left(\frac{ns}{l}\cdot\left(x-\frac{l}{2}\right)\right)}{\cosh\left(\frac{ns}{2}\right)}\right]$ (S1)

$n\approx\sqrt{\frac{2G_{\text{m}}}{E_{\text{GO}}}\left(\frac{t_{\text{GO}}}{t_{\text{m}}}\right)}$ (S2)

where *ε*_m_ is the matrix strain. *t*_GO_ and *t*_m_ are the thickness of GO and representative volume element of matrix, respectively. *l* is the size of the flake (~ 15 μm along the white dashed line in Error: Reference source not found) and *s(=l/t*_GO_*)* is the corresponding aspect ratio. *E*_GO_ is the Young’s modulus of GO, around 1/4 of that of graphene, and *G*_m_ is the shear modulus of the matrix. *x* is the position along the white dashed line in Error: Reference source not found starting from the GO edge. The value of the term *ns* can be taken as a measure of the stress transfer efficiency, and by choosing different values of *ns*, *ε*_r_ can be estimated as the function of the lateral position *x*. Since the value of *ns* of graphene has been calculated to be around 20, if the values of those parameters in Ref. are taken into calculation here, proportionally, the values of *ns* of GO can be assumed to be around 30, and the value higher than that of monolayer graphene implies a stronger interface of GO. In this situation, the ‘critical length’ can be calculated around 1~2 μm, which is smaller than that of graphene.

## 5. Definition of Stacked and Re-stacked GO structures

The thickness of a GO flake is measured by taking the height profiles of the representative section A-A’ in AFM image. For a flake where all the edges can be clearly addressed to one flake as shown in Figure S4(a), it is defined as stacked GO flake; meanwhile, if the edges can be assigned to two flakes as shown in Figure S4(b), it is defined as re-staked GO flake. Typically the number of re-stacked GO is less than 50, but it can also go down to even two re-stacked monolayer GO flakes.

**Supplementary Figure 4** The illustration of (a) stacked and (b) re-stacked GO flakes.

**References**

1. Stankovich, S, Dikin DA, Dommett GHB, Kohlhaas KM, Zimney EJ, Stach EA, Piner RD, Nguyen ST, Ruoff RS. 2006. Graphene-based composite materials. *Nature* **442,** 282-6. (doi:http://www.nature.com/nature/journal/v442/n7100/suppinfo/nature04969_S1.html)

2. Stankovich, S, Dikin DA, Piner RD, Kohlhaas KA, Kleinhammes A, Jia Y, Wu Y, Nguyen ST, Ruoff RS. 2007. Synthesis of graphene-based nanosheets via chemical reduction of exfoliated graphite oxide. *Carbon* **45,** 1558-65. (doi:http://dx.doi.org/10.1016/j.carbon.2007.02.034)

3. Yang, D, Velamakanni A, Bozoklu G, Park S, Stoller M, Piner RD, Stankovich S, Jung I, Field DA, Ventrice Jr CA, Ruoff RS. 2009. Chemical analysis of graphene oxide films after heat and chemical treatments by X-ray photoelectron and Micro-Raman spectroscopy. *Carbon* **47,** 145-52. (doi:10.1016/j.carbon.2008.09.045)

4. Jiang, T, Huang R, Zhu Y. 2014. Interfacial Sliding and Buckling of Monolayer Graphene on a Stretchable Substrate. *Advanced Functional Materials* **24,** 396-402. (doi:10.1002/adfm.201301999)

5. Gong, L, Kinloch IA, Young RJ, Riaz I, Jalil R, Novoselov KS. 2010. Interfacial Stress Transfer in a Graphene Monolayer Nanocomposite. *Advanced Materials* **22,** 2694-7. (doi:10.1002/adma.200904264)

6. Shang, J, Chen Y, Zhou Y, Liu L, Wang G, Li X, Kuang J, Liu Q, Dai Z, Miao H, Zhi L, Zhang Z. 2015. Effect of folded and crumpled morphologies of graphene oxide platelets on the mechanical performances of polymer nanocomposites. *Polymer* **68,** 131-9. (doi:http://dx.doi.org/10.1016/j.polymer.2015.05.003)

7. del Corro, E, Taravillo M, Baonza VG. 2012. Nonlinear strain effects in double-resonance Raman bands of graphite, graphene, and related materials. *Physical Review B* **85,** 033407. (doi:10.1103/PhysRevB.85.033407)

8. Young, RJ, Gong L, Kinloch IA, Riaz I, Jalil R, Novoselov KS. 2011. Strain Mapping in a Graphene Monolayer Nanocomposite. *ACS Nano* **5,** 3079-84. (doi:10.1021/nn2002079)

9. Cox, HL. 1952. The elasticity and strength of paper and other fibrous materials. *British Journal of Applied Physics* **3,** 72. (doi:10.1088/0508-3443/3/3/302 )

10. Suk, JW, Piner RD, An J, Ruoff RS. 2010. Mechanical Properties of Monolayer Graphene Oxide. *ACS Nano* **4,** 6557-64. (doi:10.1021/nn101781v)

11. Lee, C, Wei X, Kysar JW, Hone J. 2008. Measurement of the Elastic Properties and Intrinsic Strength of Monolayer Graphene. *Science* **321,** 385-8. (doi:10.1126/science.1157996)

12. Terrones, M, Martín O, González M, Pozuelo J, Serrano B, Cabanelas JC, Vega-Díaz SM, Baselga J. 2011. Interphases in Graphene Polymer-based Nanocomposites: Achievements and Challenges. *Advanced Materials* **23,** 5302-10. (doi:10.1002/adma.201102036)
