# Supplementary material for: The role of interlayer adhesion in graphene oxide upon its reinforcement of nanocomposites
Source: Philos Trans A Math Phys Eng Sci. 2016 Jul 13;374(2071):20150283. doi: 10.1098/rsta.2015.0283 (PMC4901251; doi:10.1098/rsta.2015.0283)
Supplement: Original Data Files [file rsta20150283supp2.pptx]

## Slide 1
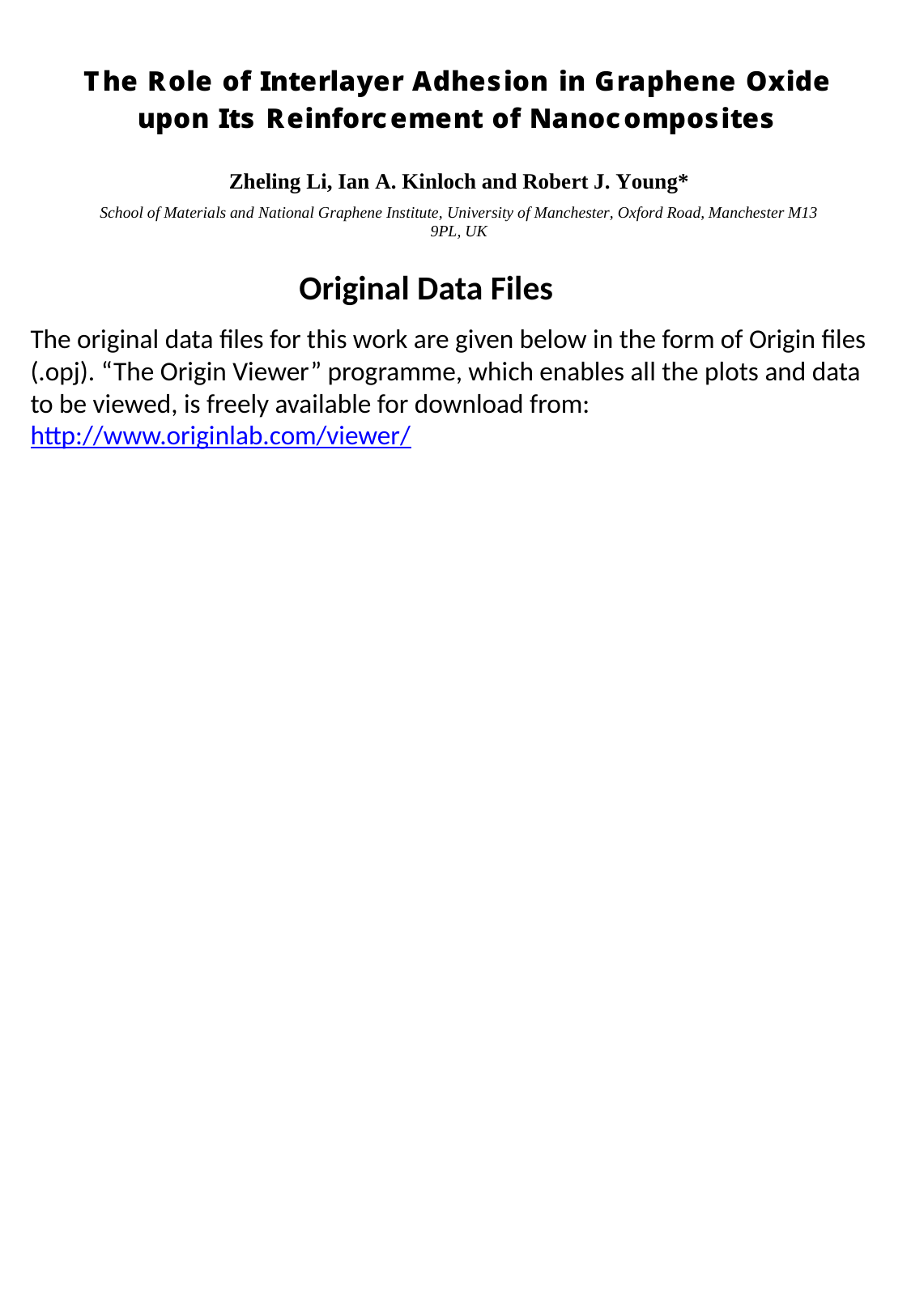

Original Data Files
The original data files for this work are given below in the form of Origin files (.opj). “The Origin Viewer” programme, which enables all the plots and data to be viewed, is freely available for download from: http://www.originlab.com/viewer/

## Slide 2
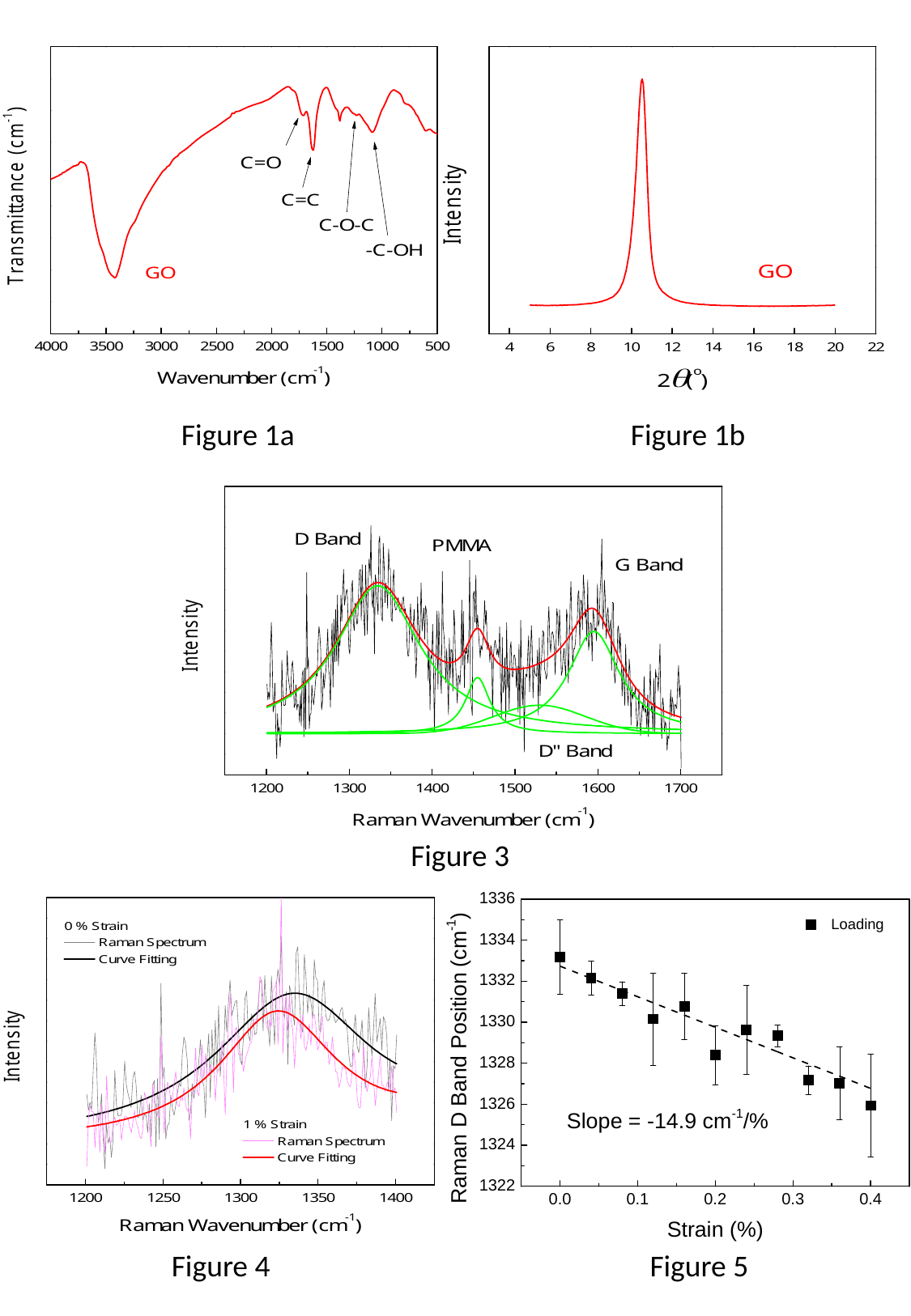

Figure 1a
Figure 1b
Figure 3
Figure 4
Figure 5

## Slide 3
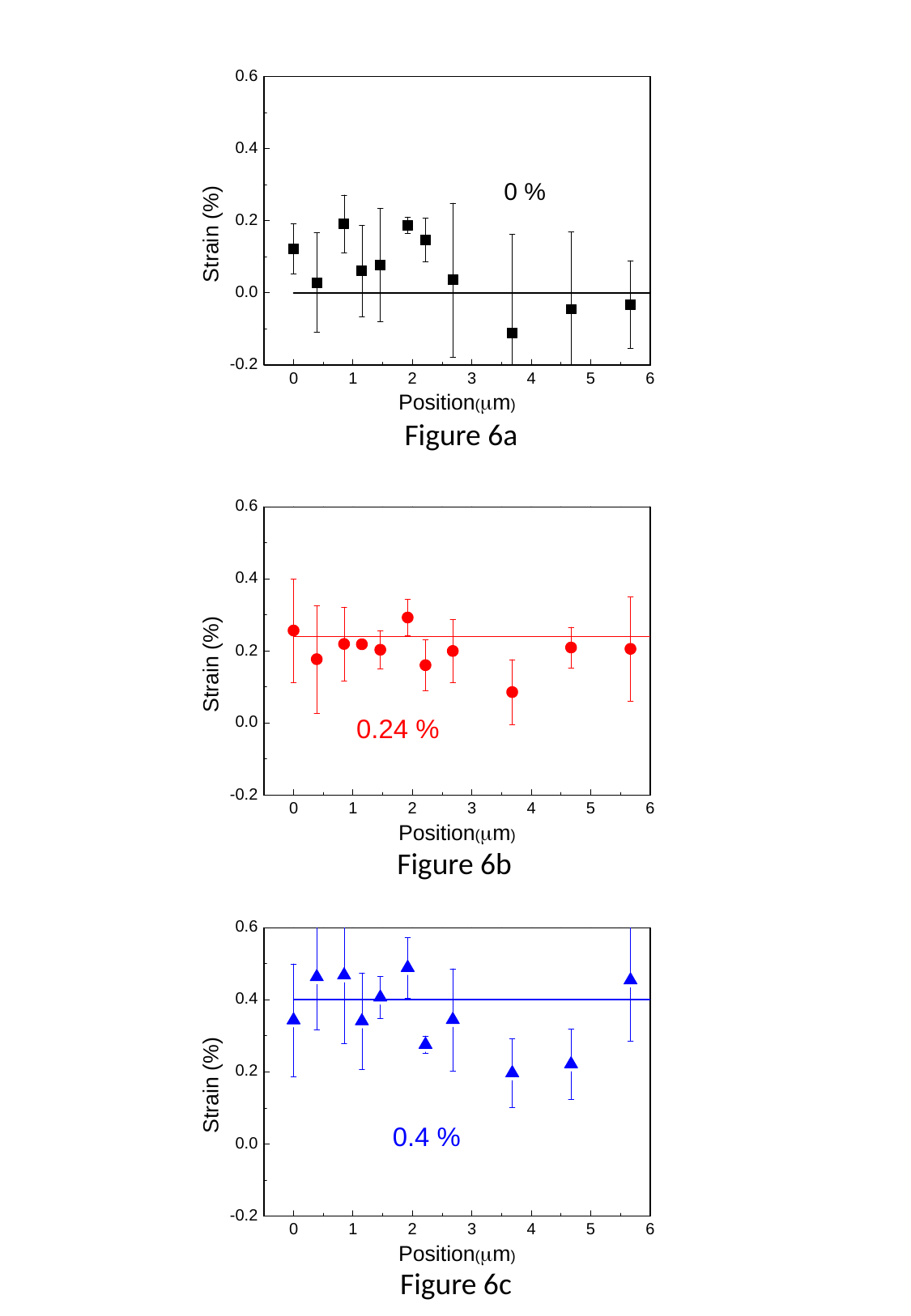

Figure 6a
Figure 6b
Figure 6c

## Slide 4
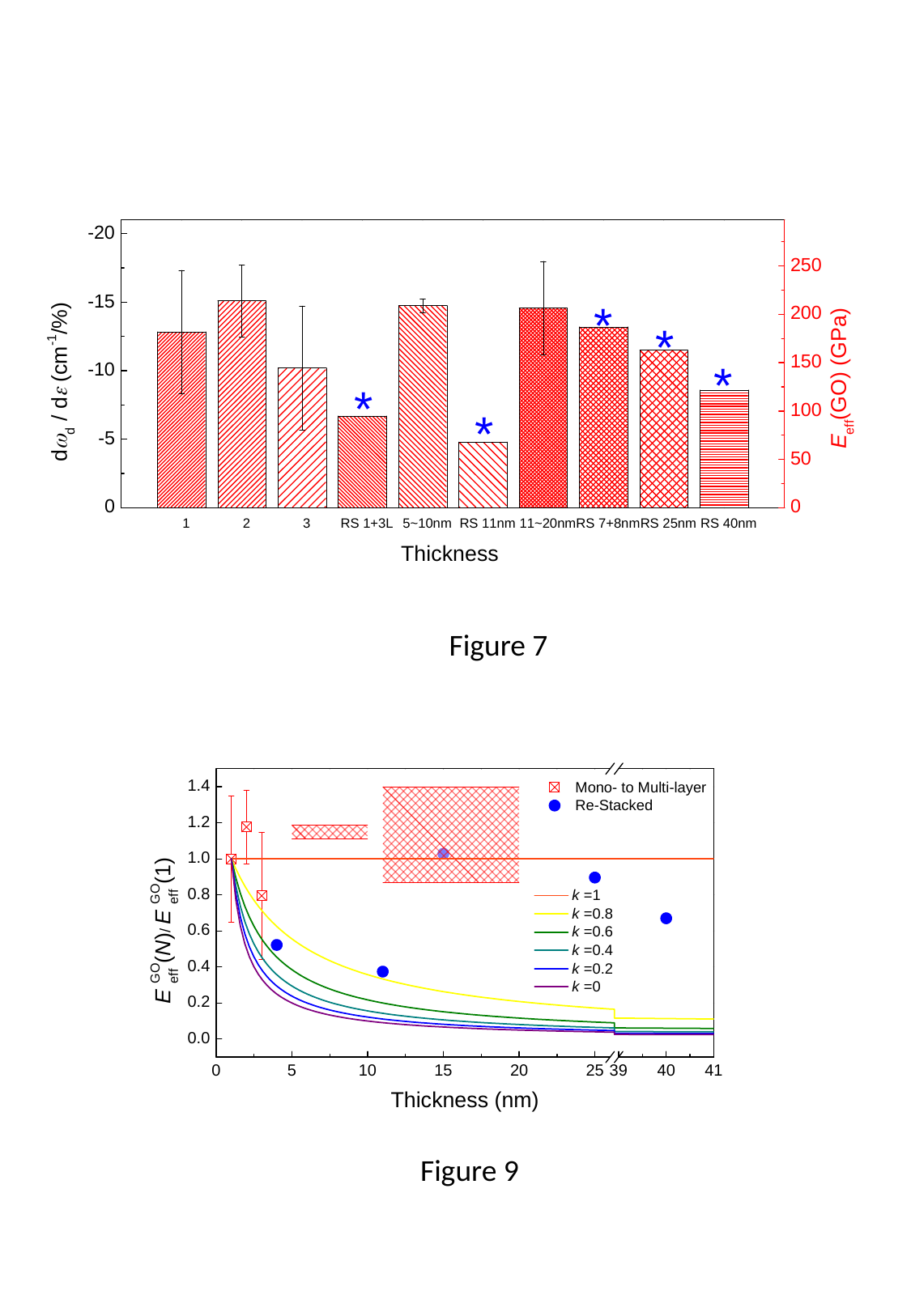

Figure 7
Figure 9

## Slide 5
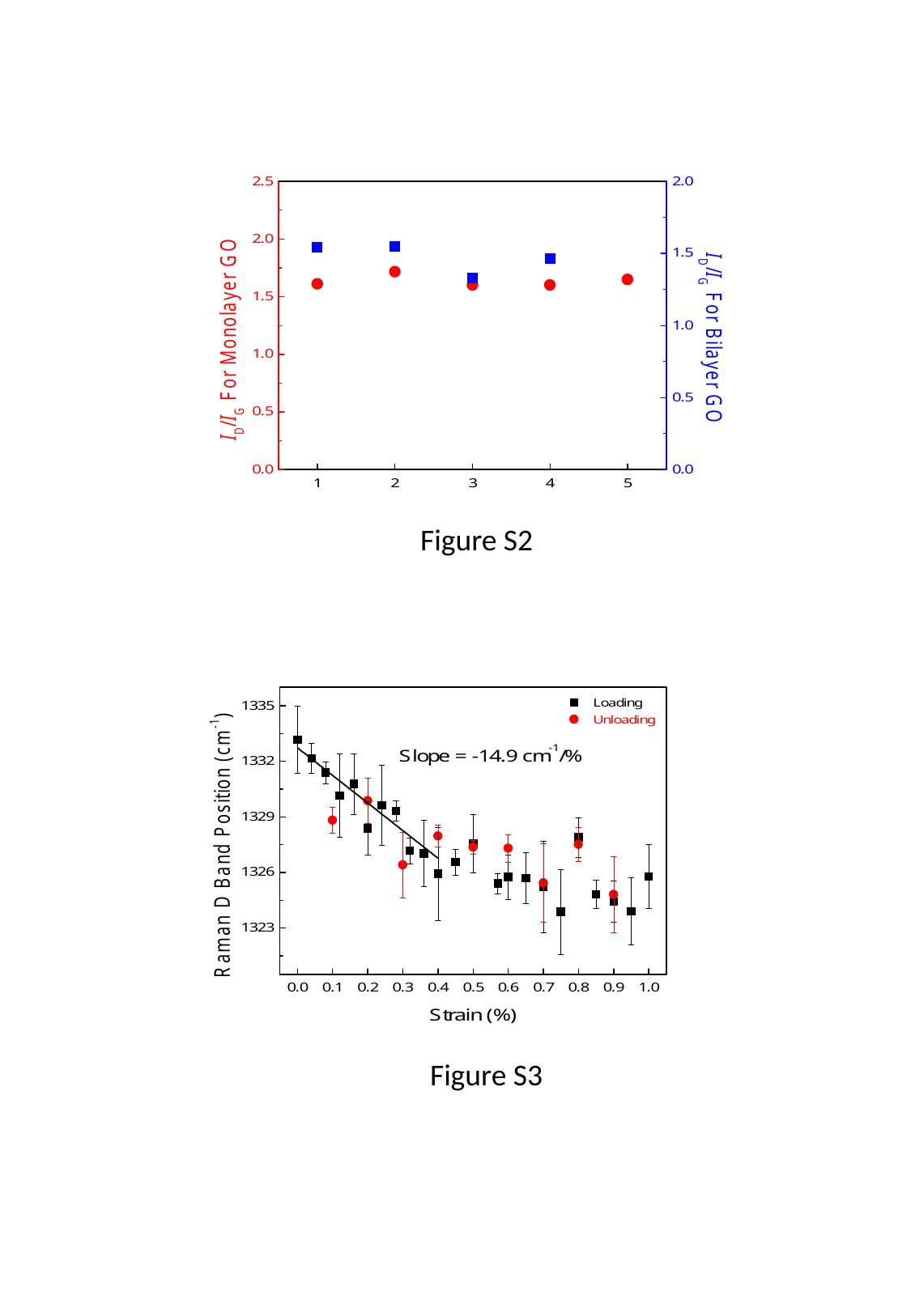

Figure S2
Figure S3
